# Injection of a PMMA‐doped MSC spheroid gel for the treatment of painful osteoporotic vertebral compression fractures

**DOI:** 10.1002/btm2.10577

**Published:** 2023-07-16

**Authors:** Wan‐Kyu Ko, Daye Lee, Seong Jun Kim, Gong Ho Han, Donghyun Lee, Seung Hun Sheen, Seil Sohn

**Affiliations:** ^1^ Department of Neurosurgery, CHA Bundang Medical Center CHA University Seongnam‐si Gyeonggi‐do Republic of Korea; ^2^ Department of Life Science CHA University Seongnam‐si Gyeonggi‐do Republic of Korea; ^3^ Preclinical Research Center Daegu‐Gyeongbuk Medical Innovation Foundation (DGMIF) Daegu Republic of Korea

**Keywords:** mesenchymal stem cell, osteoporotic fracture, pain, PMMA, spheroid

## Abstract

We aimed to develop a biocompatible treatment to overcome the limitations of polymethyl methacrylate (PMMA) vertebroplasty for osteoporotic compression fracture patients. We synthesized an injectable hydrogel containing PMMA. Mesenchymal stem cell (MSC) spheroids were included in the injectable PMMA‐doped gel (= PMMA‐doped spheroid gel). In vitro, the osteogenic/anti‐inflammatory effects of the embedded spheroids were investigated by the quantitative real‐time polymerase chain reaction method. In vivo, we used ovariectomy (OVX)‐induced osteoporotic rats with injured femurs to investigate the pain‐relief effects. The OVX rats were divided into four groups according to the materials injected (non, PMMA, PMMA gel, and PMMA‐spheroid gel) into the lesion. The immunofluorescence (IF) intensity levels of painful markers in dorsal root ganglia (DRG) were measured. In vitro, a volumetric ratio of the gel of 8 (gel):2 (PMMA) was non‐cytotoxic for MSCs and promoted the expression of osteogenic/anti‐inflammatory markers. In vivo, the values of several bone parameters in the PMMA‐doped spheroid gel group showed remarkable increases compared to those in the PMMA group. In addition, the IF intensity levels of the painful markers were noticeably decreased in the PMMA‐spheroid gel group. We, therefore, suggest that this treatment can be useful for osteoporotic vertebral compression fracture patients.

## 1 INTRODUCTION

Osteoporotic patients have low bone density levels caused by a remodeling imbalance between bone absorption and bone reconstruction.^1^, ^2^ When a bone fracture occurs in an osteoporotic patient, nociceptive signals are transferred toward the dorsal root ganglia (DRG, a cluster of neurons) attached to the spinal cord,^3^ which is followed by nociceptive and neuropathic pain.^4^, ^5^ Inflammatory cytokines resulting from the fracture also deteriorate the pain.^6^ Osteoporotic vertebral compression fractures are a major issue in clinics,^7^ as the prevalence rates of these fractures are increasing rapidly and are even highest among multiple types of osteoporotic fractures.^8^ In addition, severe pain due to the osteoporotic vertebral compression fractures may increase mortality rates.^9^

The reconstruction of collapsed bones and the inhibition of inflammatory cytokines are necessary to alleviate pain. The percutaneous vertebroplasty technique using polymethyl methacrylate (PMMA) was introduced in 1987 for pain relief.^10^ PMMA, also known as medical bone cement, is hardened by auto‐polymerization between the PMMA powder and an MMA liquid.^11^ An injection of polymerized PMMA into a lesion can alleviate pain by reconstructing the collapsed vertebra. However, this surgical therapy has two critical limitations. First, PMMA within the lesion is rarely integrated with the host bones despite the fact that organic interaction among the PMMA‐bone interfaces is essential for proper biological recovery.^12^ Second, adjacent vertebral fractures can occur due to the strength difference between the vertebral bones (10–900 MPa) and the PMMA (1700–3700 MPa).^13^ Despite these limitations, PMMA has been widely used in injections for osteoporotic vertebral compression fracture patients because biocompatible substitutes for PMMA are rare.^14^

We prepared an injectable hydrogel containing mesenchymal stem cell (MSC) spheroids and PMMA to overcome the aforementioned limitations. MSCs are promising therapeutic stem cells given their multiple capabilities, including their ability to differentiate into osteoblasts and secrete anti‐inflammatory cytokines.^15^ Transforming growth factor (TGF)‐β^16^ and interleukin (IL)‐10^17^ are two typical anti‐inflammatory cytokines secreted from MSCs. The osteogenesis and anti‐inflammatory effects of the MSC are accelerated in a three‐dimensional (3D)‐MSC spheroid structure owing to the increased number of cell–cell interactions.^18^

In our previous studies, we showed that a microporous hydrogel of glycol chitosan (gC) and oxidized hyaluronate (oHA) can offer several advantages in that it is non‐cytotoxic and fully bioresorbable.^19^, ^20^ In the present study, we used gC/oHA (CHA) gel as a carrier to embed MSC spheroids. In vitro, we added several volumes of PMMA into the CHA gel to find non‐cytotoxic and favorable viscosity for the embedded MSCs. In vivo, ovariectomy (OVX)‐induced osteoporotic femur‐injured rats were used for an evaluation of the osteogenesis/anti‐pain effects. The optimized PMMA‐doped MSC spheroid gel was injected into the injured femur. The injected PMMA‐doped spheroid gel noticeably promoted osteogenesis in the lesion. In addition, the treatment decreased the expression of the transient receptor potential vanilloid (TRPV)‐1 (a representative nociceptive ion channel marker shown in neurons) and the ionized calcium binding adaptor molecule (iba)‐1 (a representative microglial marker activated by inflammatory stimulation) in DRGs.^21^

## 2 RESULTS

### 2.1 Characteristics/cytotoxicity according to the volumetric ratio between the gel and PMMA

**FIGURE 1:**
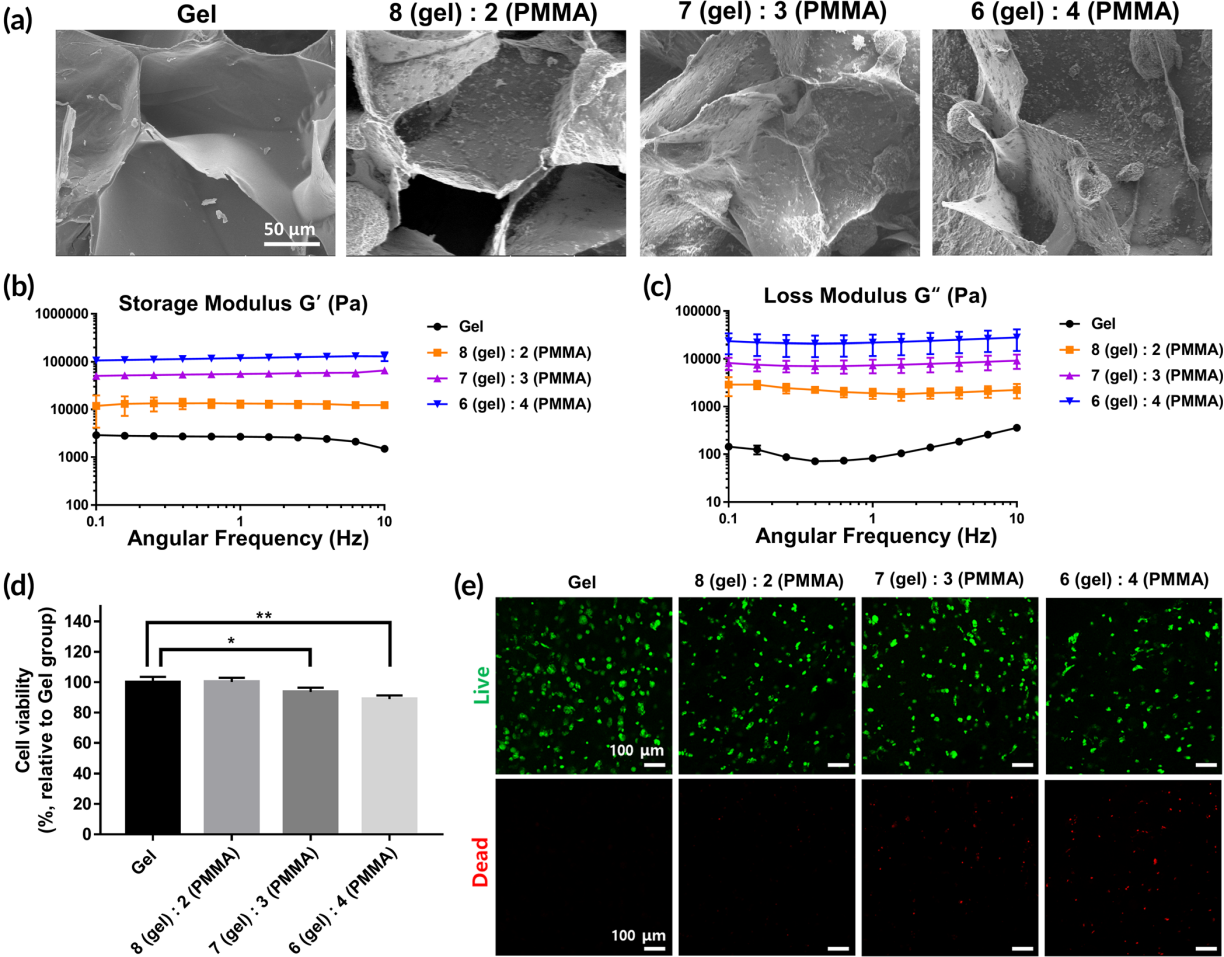
Characteristics and cytotoxicity test. (a) observation of the gel structures using scanning electron microscopy at several volumetric ratios of polymethyl methacrylate (PMMA)‐doped gels. Rheological properties of (b) the storage modulus (G′) and (c) loss modulus (G″) at several volumetric ratios. Cell viability tests at several volumetric ratios of gels embedding mesenchymal stem cells (MSCs) using a (d) cell counting kit and (e) a Live&Dead staining kit.

Gel forming process between gC and oHA was provided in Figure S1. The morphologies at several volumetric ratios of gel and PMMA (8:2, 7:3, and 6:4) are presented as scanning electron microscope (SEM) images (Figure 1a). Rheological properties at each ratio are shown in Figure 1b (for storage modulus G′) and Figure 1c (for loss modulus G″). At 10 Hz, the G′ value was 1.5 ± 0.1 kPa in the Gel group. Among the PMMA‐mixed groups, the G′ values were increased in the order of the 8:2 (12.3 ± 1.8 kPa), 7:3 (65.7 ± 6.8 kPa), and 6:4 (130.7 ± 27.3 kPa) groups.

As shown in Figure 1d, the cell viability rates in the gel and 8 (gel):2 (PMMA) groups were 100 ± 3.5% and 100.2 ± 2.7%, respectively. However, the rates were decreased in the order of the 7:3 (93.5 ± 2.9%) and 6:4 (88.9 ± 2.4%) groups. Indeed, we could not observe dead cells in the gel and 8:2 groups, whereas dead cells were found in the 7:3 and 6:4 groups (Figure 1e). We chose the gel with the 8:2 ratio as the scaffold for embedding the MSC spheroids. We measured the gel mass of the 8:2 ratio for 21 days (Figure S2). The mass of the remaining gel was 51.4 ± 3.3%, 29.8 ± 2.7%, and 6.6 ± 0.9% at 7, 14, and 21 days, respectively.

### 2.2 MSC spheroid creation and quantitative real‐time polymerase chain reaction/enzyme‐linked immunosorbent assay evaluation in vitro

**FIGURE 2:**
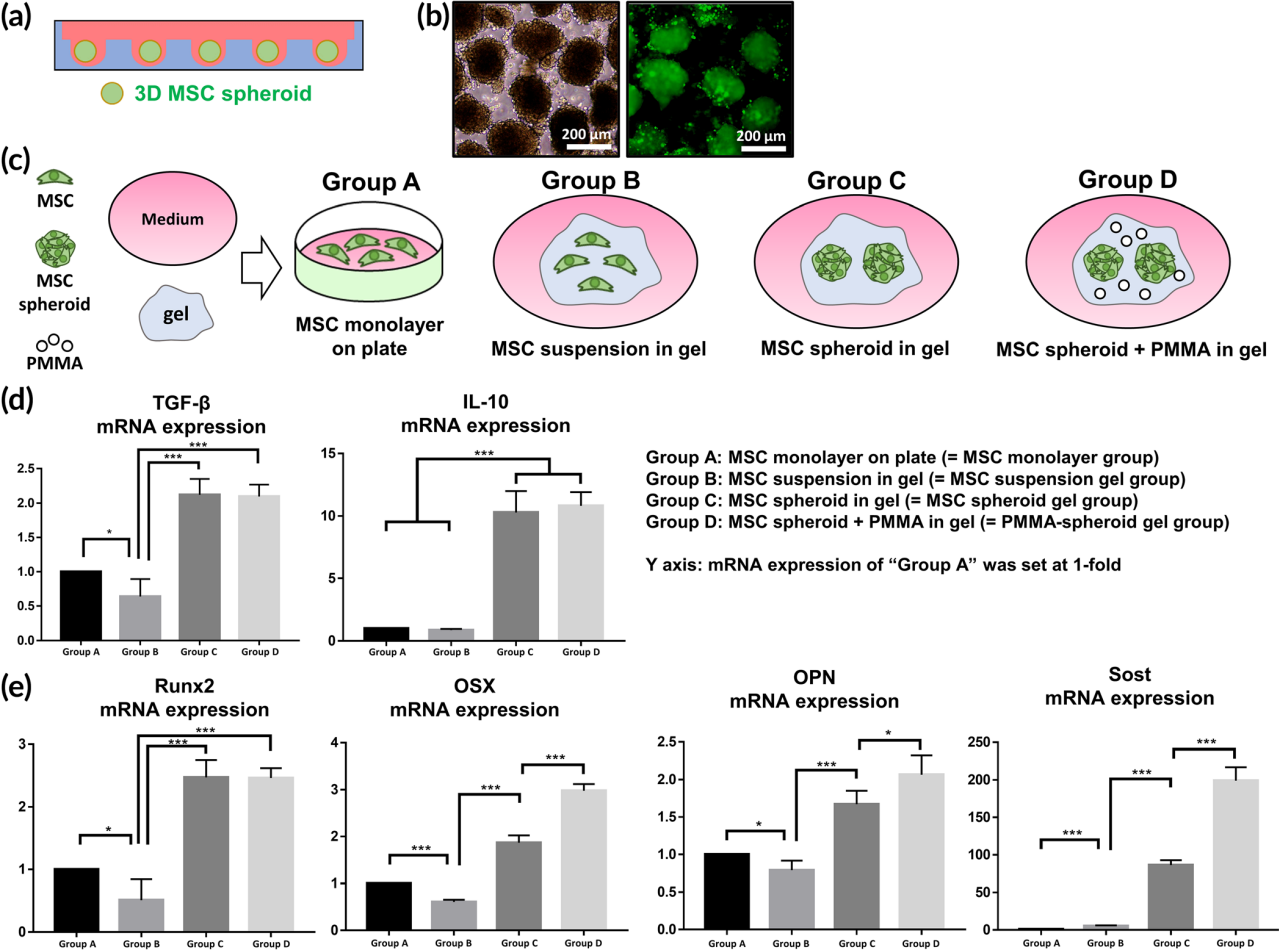
In vitro evaluation. (a) three‐dimensional (3D) MSC spheroids. (b) Observation of the synthesized 3D MSC spheroid (scale bar: 200 μm). (c) Four types of experimental groups (MSC monolayer on a plate, MSC suspension in the gel, MSC spheroid in the gel, and PMMA + MSC spheroid in the gel) for the in vitro evaluation. Cell pellets were subjected to a quantitative real‐time polymerase chain reaction (qRT‐PCR) analysis to detect the mRNA values for (d) transforming growth factor (TGF)‐β, interleukin (IL)‐10, (e) runt‐related transcription factor 2 (Runx2), osterix (OSX), osteopontin (OPN), and sclerostin (Sost) genes. Multiple comparisons among the four groups were conducted with a one‐way analysis of variance (ANOVA). The results are expressed as the mean ± standard error of the mean (SEM, n = 4 per group): *p < 0.05, **p < 0.01, and ***p < 0.001; significant differences among the four groups were shown. MSC, mesenchymal stem cell; PMMA, polymethyl methacrylate.

The diameter of an MSC spheroid (Figure 2a) as created here was approximately 200 μm (Figure 2b). We investigated whether the PMMA added to an MSC spheroid‐embedded gel (= Group D: PMMA‐spheroid gel group) would induce the osteogenesis/secretion of anti‐inflammatory cytokines. For a specific evaluation, we added three control groups (Group A: MSC monolayer group, Group B: MSC suspension gel group, and Group C: MSC spheroid gel group; Figure 2c). As shown in Figure 2d, the values of TGF‐β mRNA in Group C (2.12 ± 0.23, ***p < 0.001) and Group D (2.10 ± 0.17; ***p < 0.001) were significantly increased compared to those in Group A (1.00 ± 0.00) and Group B (0.64 ± 0.26). The IL‐10 mRNA values were also significantly increased in the two groups (Group C: 10.28 ± 1.72, ***p < 0.001 and Group D: 10.82 ± 1.10, ***p < 0.001) compared to those in Group A (1.00 ± 0.00) and Group B (0.87 ± 0.10). As shown in Figure S3a (for TGF‐β) and Figure S3b (for IL‐10), an increasing tendency in Groups C and D was also shown in the protein levels. The secreted TGF‐β proteins amounted to 78.93 ± 8.18 pg/mL (Group A), 75.08 ± 7.74 pg/mL (Group B), 118.81 ± 6.98pg/mL (Group C), and 116.37 pg/mL ± 9.44 (Group D). However, we could not find any significance between Groups C and D (Figure 2d and Figure S3).

We investigated typical osteogenesis‐related genes, in this case, runt‐related transcription factor 2 (Runx2), osterix (OSX), osteopontin (OPN), and sclerostin (Sost) (Figure 2e). The level of osteogenic mRNA as well as that of anti‐inflammatory TGF‐β/IL‐10 mRNA was significantly increased in Groups C and D compared to those in Groups A and B (***p < 0.001). In addition, the values in Group D were significantly higher compared to those in Group C in the OSX (***p < 0.001), OPN (*p < 0.05), and Sost (***p < 0.001) cases.

### 2.3 OVX‐induced osteoporosis and osteogenic/anti‐pain effects evaluation in vivo

**FIGURE 3:**
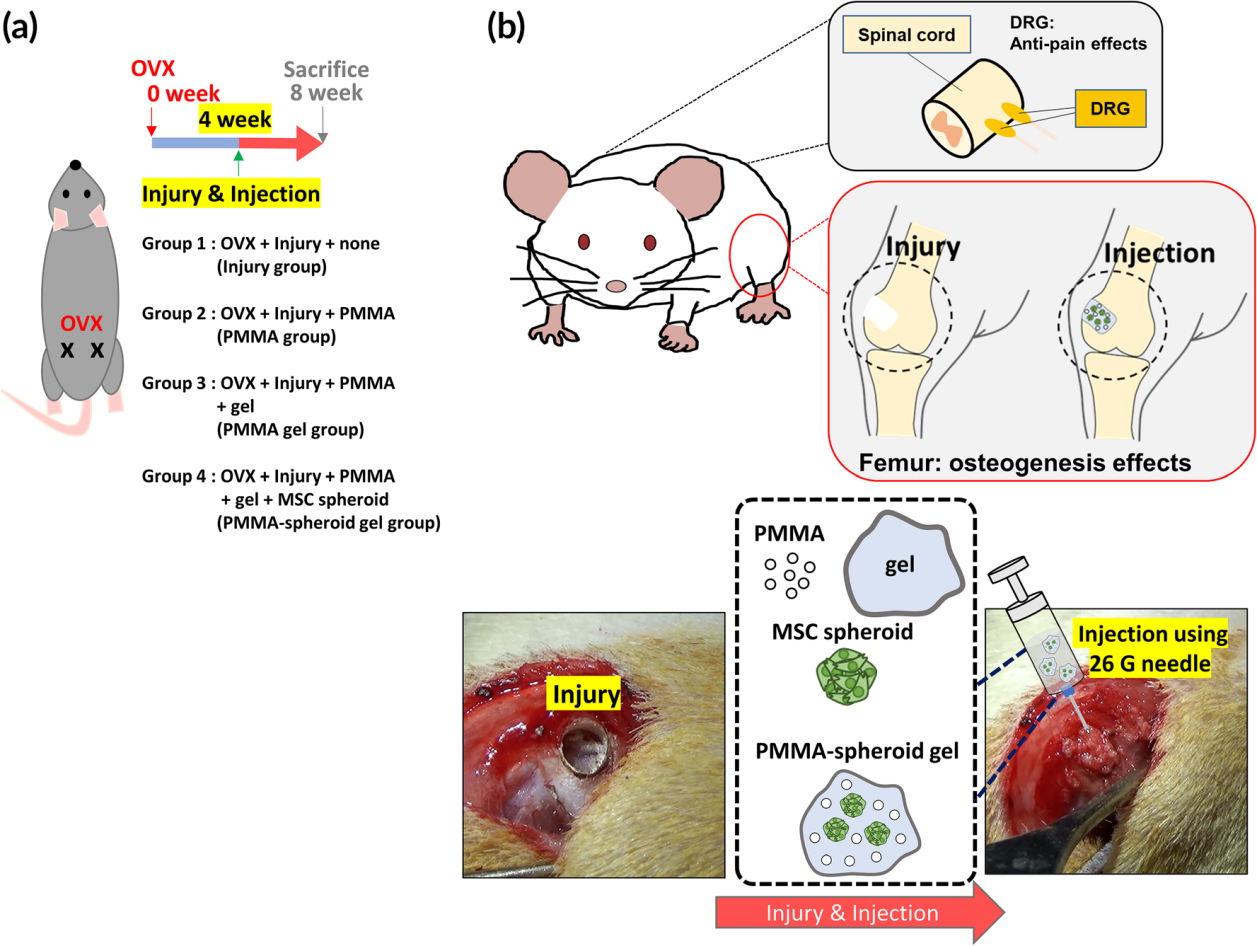
Schematic design for the in vivo evaluation. (a) an ovariectomy (OVX) was conducted before the femur injuries for osteoporosis modeling in Sprague–Dawley rats. The rats were divided into four experimental groups according to the injected material. Group 1: Injury group (OVX + Injury + none), Group 2: PMMA group (OVX + Injury + PMMA), Group 3: PMMA gel group (OVX + Injury + PMMA + gel), and Group 4: PMMA‐spheroid gel group (OVX + Injury + PMMA + gel + MSC spheroid). Four weeks after OVX, each material was injected into the lesion. (b) Four weeks after these injections, all rats were sacrificed. Femurs and dorsal root ganglia (DRG) were extracted for an in vivo evaluation. PMMA, polymethyl methacrylate.

We also studied whether the PMMA‐spheroid gels implanted into the injured femurs could promote osteogenesis for pain relief. The schematic design for these in vivo experiments is shown in Figure 3a. In addition to the PMMA‐spheroid gel group (Figure 3b), three control groups (Injury, PMMA, and PMMA gel groups) were included for the in vivo evaluation.

**FIGURE 4:**
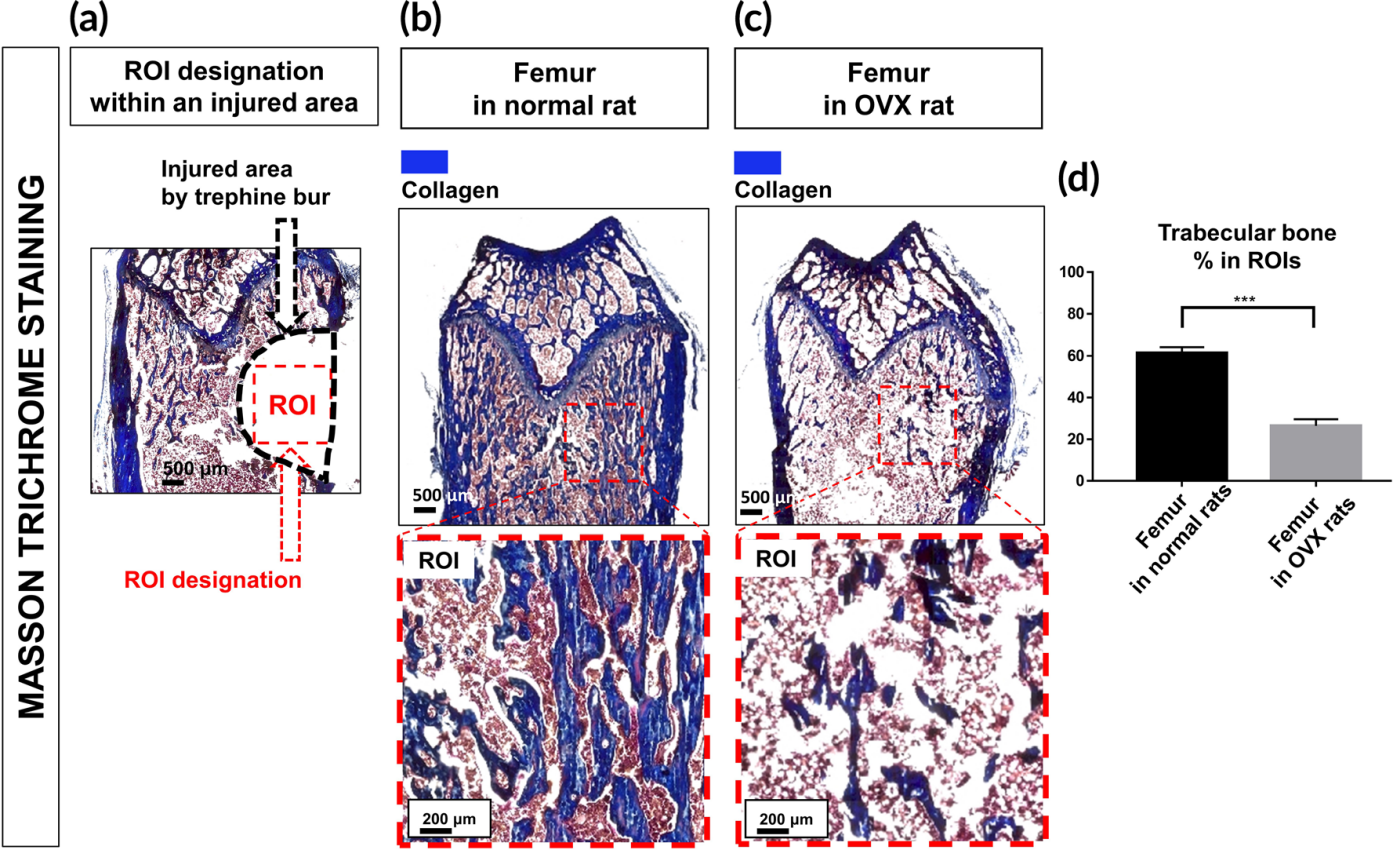
Osteoporotic evaluation using Masson trichrome staining (MTS). Collagen in the femur is shown to be stained in blue. (a) A region of interest (ROI, 1800 × 1800 μm^2^) was designated within the injured area in each case. Representative images of (b) a normal femur and (c) an OVX‐induced osteoporotic femur are shown. (d) Trabecular bones in the ROIs were quantified. The results are expressed as the mean ± SEM (*n* = 6 per group). “***” indicates a significant difference for *p* < 0.001. OVX, ovariectomy; ROI, region of interest.

Before the osteogenic evaluation, we investigated whether osteoporosis occurred in the OVX rats (Figure 4). The corresponding images are shown in Figure 4a (for region of interest [ROI] designation), Figure 4b (for normal rats), and Figure 4c (for OVX rats). As shown in Figure 4d, the trabecular bone areas were significantly decreased in the OVX rats (26.47 ± 3.18%, ***p < 0.001) compared to that in the normal rats (61.37 ± 2.84%).

**FIGURE 5:**
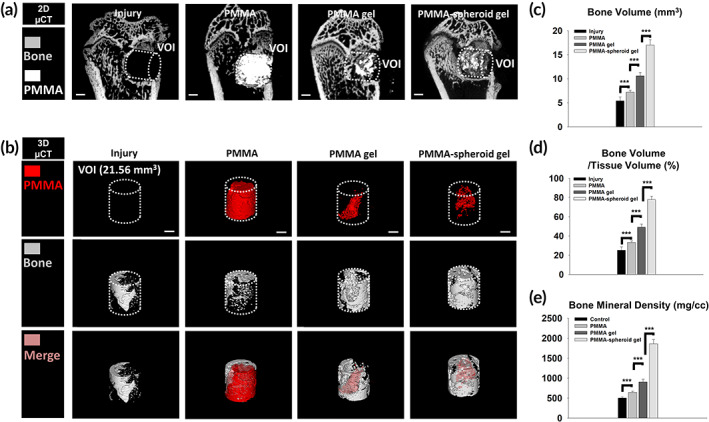
Osteogenic evaluation. Representative (a) 2D and (b) 3D images were reconstructed by micro‐computed tomography (μCT) equipment (scale bar: 1 mm). Quantification was performed at the volume of interest (VOI, 21.56 mm^3^) for (c) the bone volume (BV, mm^3^), (d) BV/tissue volume (TV, %), and (e) bone mineral density (BMD, mg/cc). The results are expressed as the mean ± SEM (*n* = 6 per group). “***” indicates a significant difference for *p* < 0.001.

The femurs in the Injury, PMMA, PMMA gel, and PMMA‐spheroid gel groups were reconstructed as 2D‐micro‐computed tomography (μCT; Figure 5a) and 3D‐μCT images (Figure 5b). The regenerated bone volume (BV) values within a volume of interest (VOI) were 5.5 ± 1.0 mm^3^ (25.2 ± 3.5%), 7.2 ± 0.6 mm^3^ (33.3 ± 1.8%), 10.6 ± 0.7 mm^3^ (49.0 ± 3.3%), and 17.0 ± 1.1 mm^3^ (77.8 ± 3.4%) in the Injury, PMMA, PMMA gel, and PMMA‐spheroid gel groups, respectively (Figure 5c,d, ***p < 0.001). The bone mineral density (BMD) values were also significantly (***p < 0.001) increased in the order of the Injury (503.2 ± 27.0 mg/cc), PMMA (646.9 ± 28.3 mg/cc), PMMA gel (906.1 ± 68.3 mg/cc), and PMMA‐spheroid gel groups (1861.1 ± 105.4 mg/cc; Figure 5e). In other words, osteogenesis within the injured bone was most strongly accelerated in the PMMA‐spheroid gel group.

**FIGURE 6:**
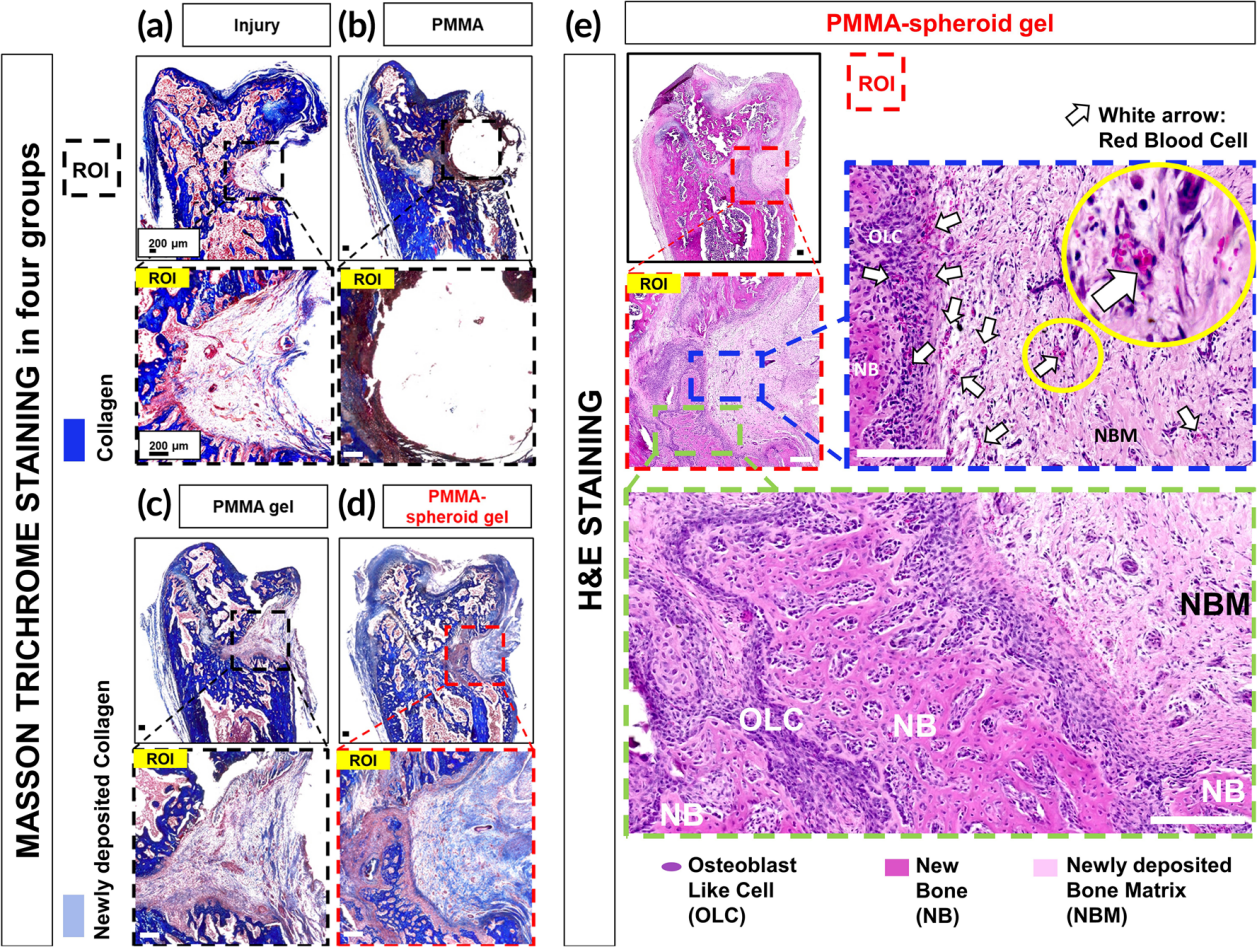
Histological evaluation. Representative MTS images for the (a) Injury, (b) PMMA, (c) PMMA gel, and (d) PMMA‐spheroid gel groups (scale bar: 200 μm). An ROI (1800 × 1800 μm^2^) per femur is designated. (e) Representative hematoxylin and eosin (H&E)‐stained images for the PMMA‐spheroid gel group (scale bar: 200 μm). Blue, light blue, purple, deep pink, light pink, and red in the ROI indicate collagen, newly deposited collagen, osteoblast‐like cells (OLCs), new bone (NB), newly deposited bone matrix (NBM), and red blood cells (RBCs, designated by the arrows), respectively. MTS, Masson trichrome staining; PMMA, polymethyl methacrylate; ROI, region of interest.

For a histological assessment, we stained the femurs with Masson trichrome staining (MTS) kits. As shown in Figure 6a (for the Injury group), Figure 6b (for the PMMA group), Figure 6c (for the PMMA gel group), and Figure 6d (for the PMMA‐spheroid gel group), the collagen‐stained areas are expressed as blue. In the PMMA group, we found that most of the areas in the ROI were empty (Figure 6b). However, the injured areas were mostly filled with collagen in the PMMA‐spheroid gel group (Figure 6d). For a further specific histological analysis of the PMMA‐spheroid gel group, we stained the femurs with a hematoxylin and eosin (H&E) solution. The typical bone parameters, osteoblast‐like cells (OLCs, stained as purple), new bone (NB, stained as deep pink), and newly deposited bone matrix (NBM, stained as light pink), were demonstrated in the ROI (Figure 6e). Red blood cells (RBCs, stained as red) appeared locally at several sites (as indicated by the arrows) in the PMMA‐spheroid gel group. The bone parameters were also determined in the Injury (Figure S4a), PMMA (Figure S4b), and PMMA gel (Figure S4c) groups. However, two parameters, NB and RBC, were most abundant in the PMMA‐spheroid gel group among the four groups (Figure 6e and Figure S4).

**FIGURE 7:**
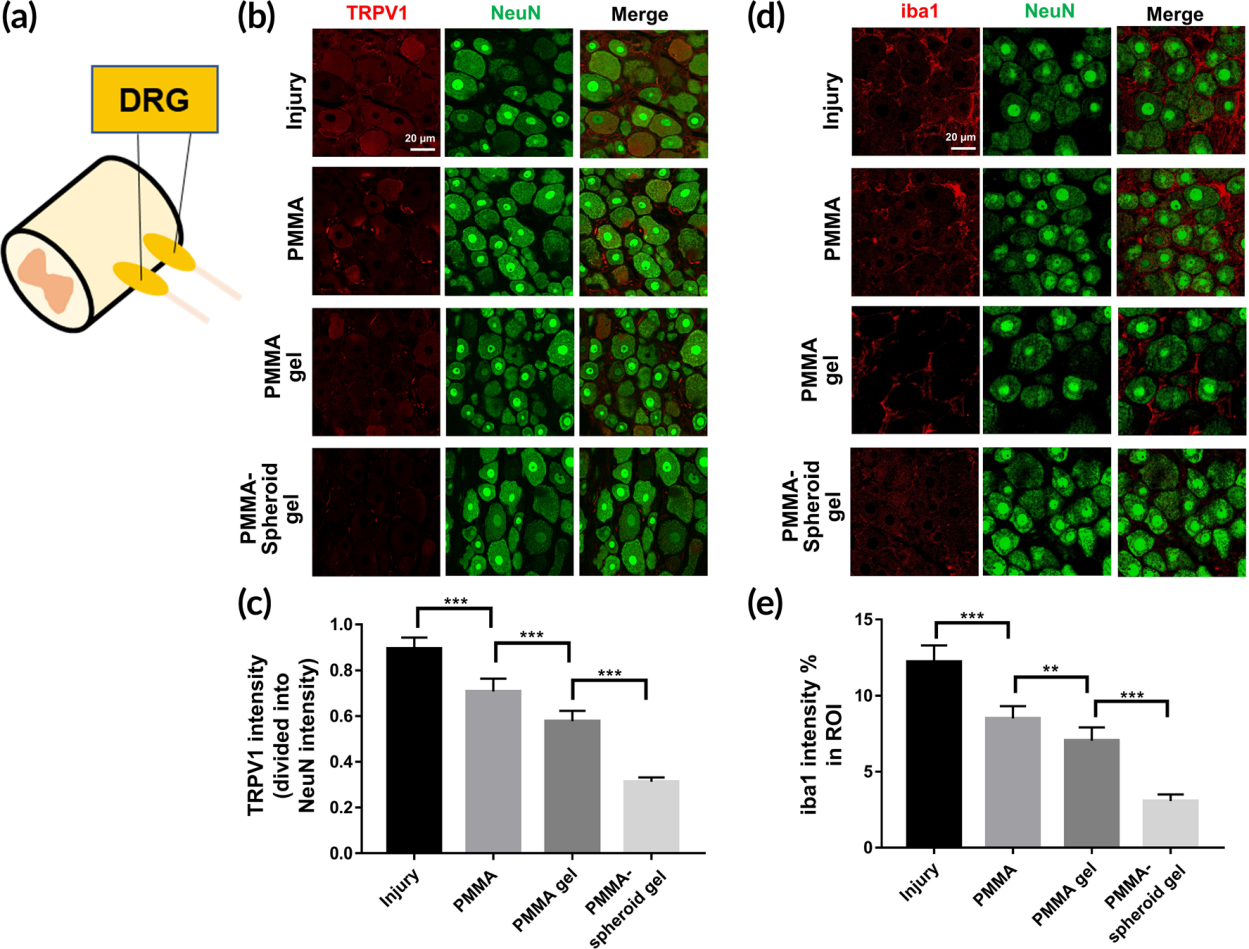
Immunofluorescence (IF) staining in (a) DRG. (b) transient receptor potential vanilloid (TRPV1)/neuronal nuclei (NeuN) staining (ROI: 200 × 200 μm^2^, scale bar: 20 μm). (c) TRPV1 quantification (*n* = 8 per group). (d) Ionized calcium binding adaptor molecule (iba)1/NeuN staining (ROI: 200 × 200 μm^2^, scale bar: 20 μm). (e) iba1 quantification. Results are the mean ± SEM: **p < 0.01, and ***p < 0.001; significant differences among the four groups were demonstrated. DRG, dorsal root ganglia; ROI, region of interest.

We stained the DRGs (Figure 7a) with TRPV1/neuronal nuclei (NeuN) (Figure 7b,c) or iba1/NeuN (Figure 7d,e) markers. The TRPV1 intensity levels were gradually decreased in the order of the Injury (0.89 ± 0.05), PMMA (0.71 ± 0.06), PMMA gel (0.58 ± 0.05), and PMMA‐spheroid gel (0.31 ± 0.02) groups (Figure 7c, ***p < 0.001). This gradually decreasing tendency was also found in the iba1 marker (Figure 7d). The iba1 intensity levels (%) in the ROIs were 12.21 ± 1.09%, 8.50 ± 0.81%, 7.04 ± 0.87%, and 3.07 ± 0.44% for the Injury, PMMA, PMMA gel, and PMMA‐spheroid gel groups, respectively (Figure 7e, **p < 0.01).

## 3 DISCUSSION

In this study, we focused on overcoming the two critical limitations (adjacent vertebral fracture possibility and osteogenesis without organic interaction) of percutaneous PMMA therapy (vertebroplasty) for osteoporotic compression fracture patients. We prepared a PMMA additive gel containing MSC spheroids. The implanted PMMA‐spheroid gel promoted osteogenesis with organic interaction in lesions. In addition, the empty space due to degraded gels was filled with regenerated bone tissue.

The microporous gel used in this study enabled the embedding of PMMA (Figure 1a). Among several ratios, the 8 (gel):2 (PMMA) volumetric ratio of the gel was the optimal scaffold for MSC spheroid embedding. The ratio was non‐toxic for MSCs (Figure 1d,e). The additive PMMA did not interfere with the TGF‐β/IL‐10 expression levels from the MSC spheroids (Figure 2d and Figure S3). Moreover, the ratio is helpful for osteogenesis of the embedded MSC spheroids (Figure 2e). The viscosity, at 12.3 ± 1.8 kPa, of the 8:2 ratio of gel may induce the osteogenesis of the embedded spheroids. According to work by He et al., viscosity of 1–60 kPa is advantageous for the osteogenesis of MSCs.^22^ Huebsch et al. also showed that the osteogenic differentiation of MSCs was induced in 11–30 kPa of a matrix.^23^ These results indicate that the osteogenic differentiation of MSCs is affected by the environment. OSX is a major marker of pre‐osteoblasts.^24^ OPN^25^ and Sost^26^ are expressed in mature osteoblasts. We found that OSX/OPN/Sost mRNA were significantly increased with the viscosity of the PMMA‐spheroid gel in vitro (Figure 2e).

MSC spheroids have been widely implanted into degenerative bones because the spheroids can directly contribute the bone regeneration.^27^ We investigated the osteogenic/anti‐pain effects of the PMMA‐spheroid gel using OVX‐induced osteoporotic femur‐injured rats (Figure 3). The removal procedure of two ovaries has been widely conducted for osteoporotic modeling in rats.^15^, ^28^ Researchers have measured trabecular bones in femurs for osteoporosis evaluations. Collagen is the most abundant matrix in trabecular bones,^29^ Considering that vertebral compression fractures mainly result from attenuated densities of trabecular bones in osteoporotic patients,^30^ trabecular bones composed of abundant collagen can be the major indicator during an osteoporotic evaluation. We sacrificed the rats 8 weeks after the OVX procedure found that the ratio of trabecular bones was significantly decreased in the OVX rats compared to the normal rats (Figure 4d).

In this study, we used osteoporotic rats with injured femurs instead of patients with osteoporotic vertebra compression fractures for pain modeling. In both rat femurs and human spines, a considerable amount of tissue is composed of trabecular bone.^31^ Several researchers have shown that femur‐injured rats can be a pain‐inducing model, as the pain signals resulting from the injured femur are transferred to the DRGs and activate the TRPV1/iba1 expression in osteoporotic rats.^32^, ^33^ In addition, inflammatory responses due to the injured femur accelerate nociceptive/neuropathic pain by activating the TRPV1/iba1 expression in DRGs.^34^ We found that the expression levels of TRPV1 (Figure 7c) and iba1 (Figure 7e) were highest in the Injury group.

Mixed materials, including PMMA/cells, are rarely used as a biocompatible treatment because the organic interaction between the PMMA and the cells is quite poor.^12^ Indeed, we found that most ROI regions were empty in the PMMA group (Figure 6b and Figure S4b). The poor interaction between the host cells and the implanted PMMA may cause such empty spaces. In contrast, we showed that MSC spheroids embedded at an appropriate volumetric ratio (in this case, 8 (gel):2 (PMMA)) of a PMMA‐doped gel can promote osteogenesis with organic interaction (Figures 5 and 6e). During osteogenesis, angiogenesis resulting from RBCs is a crucial factor related to biological bone healing.^35^, ^36^ The relatively abundant RBC expression in the PMMA‐spheroid gel group (= treatment group, Figure 6e) compared to those in the other three control groups (Figure S4) indicates that biocompatible osteogenesis occurred due to the implanted PMMA‐MSC spheroid gel. However, we could not distinguish the osteogenesis from the implanted MSCs and host MSCs. Considering that host MSCs rarely migrate toward the lesion for bone reconstruction when osteoporosis occurs,^15^ the highest osteogenic outcomes and the lowest expression levels of the pain markers in the treatment group may have resulted from the implanted MSC spheroids. In other words, the implanted PMMA‐doped spheroid gel contributed to osteogenesis in the lesion (Figures 5 and 6e), leading to anti‐pain effects in the DRGs (Figure 7).

## 4 MATERIALS AND METHODS

### 4.1 Materials

Rat‐bone‐marrow‐derived MSCs and Live&Dead staining kits were purchased from Invitrogen Life Sciences. The 6/48‐well cell culture plates used in this study were obtained from Falcon Becton Dickinson (Falcon). Deionized water (18.2 MΩ) was prepared using an EXL‐3 water purification system (Vivagen). The PMMA kit (Spinefix^R^) was purchased from Teknimed SAS.

### 4.2 Preparation of the PMMA, gel, and the gels containing PMMA

PMMA powder and MMA liquid were mixed according to the manufacturer's instructions for polymerization.^37^ CHA gels were prepared as previously described.^19^ Detailed preparation methods for CHA gels and PMMA are given in the Supporting Information. The CHA gel was mixed with the completely polymerized PMMA to prepare PMMA‐doped gels with volumetric ratios of 8 (gel):2 (PMMA), 7 (gel):3 (PMMA), and 6 (gel):4 (PMMA).

### 4.3 SEM and rheological properties

Four gel samples with different ratios (Gel [300 μL], 8 [gel 240 μL]:2 [PMMA 60 μL], 7 [gel 210 μL]:3 [PMMA 90 μL], and 6 [gel 180 μL]:4 [PMMA 120 μL]) were prepared for SEM imagery and rheological analyses. The SEM images were taken by an SU8010 device (HITACHI) at 500× magnification. The frequency‐dependent storage modulus (G′) and loss modulus (G″) in the gels were measured up to 10 Hz using a rotating rheometer (*n* = 4 per group, MCR 92, Anton‐Paar).

### 4.4 Gel degradation test of 8 (gel):2 (PMMA) ratios

The mass of the 8 (gel 32 μL):2 (PMMA 8 μL) ratio was measured at 7, 14, and 21 days (*n* = 3). They were incubated in DPBS (37°C, 100 rpm) and the DPBS was refreshed every 3 days. The initial gel weight was designated as 100% and the weight of the remaining gel was also demonstrated as %.

### 4.5 Cell viability test

MSCs were proliferated and sub‐cultured with a MesenPRO RS™ basal medium kit (Thermo Fisher Scientific) supplemented with 1% penicillin–streptomycin (GIBCO) for in vitro/vivo experiments. MSCs (5 × 10^5^) were embedded in the gels with four volumetric ratios (Gel [50 μL], 8 [gel 40 μL]:2 [PMMA 10 μL], 7 [gel 35 μL]:3 [PMMA 15 μL], and 6 [gel 30 μL]:4 [PMMA 20 μL]). Afterward, they were placed on 48‐well cell culture plates and incubated with the medium for 2 days (*n* = 4 per group). A cytotoxicity test was conducted using a cell counting kit (EZ‐Cytox, Daeil Labservice). The absorbance in the gel group was fixed at 100% and the relative absorbance levels in the other groups were calculated. Under identical conditions, the gels with the four ratios were stained with calcein‐AM (to stain living cells) and ethidium homodimer‐1 (EthD‐1, to stain dead cells). Detailed descriptions of the cell viability/staining tests are provided in the Supporting Information.

### 4.6 MSC spheroid preparation and observation

MSCs (1 × 10^6^) were seeded into concave microwells (StemFIT 3D, #H389600L, MicroFIT) and incubated for 24 h to form MSC spheroids. The thus‐created MSC spheroids were observed at 10× magnification through an optical microscope (Olympus IX71) and a confocal laser‐scanning microscope (LSM 880, Carl Zeiss).

### 4.7 qRT‐PCR and ELISA analyses in vitro

MSCs were seeded onto a six‐well cell culture plate (= Group A). We prepared the following four groups: Group A: MSC monolayer (1 × 10^6^) group, Group B: MSC suspension (1 × 10^6^) gel (40 μL) group, Group C: MSC spheroid (1 × 10^6^) gel (40 μL) group, and Group D: PMMA (8 μL)‐spheroid (1 × 10^6^) gel (32 μL) group. They were cultured in the medium for 7 days (*n* = 4 per group). The total RNA of the embedded MSCs was isolated using TRIzol reagent (Invitrogen), as previously described.^38^ The RNA was synthesized to complementary DNA (cDNA) for the detection of TGF‐β, IL‐10, Runx2, OSX, OPN, and Sost genes. The relative values of the target genes were normalized to glyceraldehyde 3‐phosphate dehydrogenase (GAPDH) using the $2^{-\Delta \Delta C_{T}}$ method.^39^ Detailed methods and the primer sequences (Table S1) for the quantitative real‐time polymerase chain reaction (qRT‐PCR) analyses are given in the Supporting Information. We also measured the TGF‐β/IL‐10 proteins secreted from the embedded MSCs using enzyme‐linked immunosorbent assay (ELISA) kits (Koma Biotech). The values of the secreted proteins were measured based on the manufacturer's instructions.^40^ The qRT‐PCR and ELISA experiments were conducted in triplicate.

### 4.8 Osteoporosis modeling

Eight‐week‐old female Sprague–Dawley rats (Raonbio Inc.) weighing between 170 g and 210 g were anesthetized by an intraperitoneal administration of a combination of Rompun (10 mg/kg, Bayer Animal Health Co.) and Zoletil (50 mg/kg, Virbac Laboratories). The rats were ovariectomized and housed for 8 weeks under the following conditions: room temperature (20–23°C), 55% humidity, and a 12 h circadian light rhythm with free access to water and food.

### 4.9 Four experimental groups for in vivo evaluation

Four weeks from the OVX, the distal parts of the femurs were injured in a cylinder form (diameter: 2.7 mm, depth: 3 mm) by a circular trephine burr (Fine Science Tools). Afterward, three types of treatments were injected into the injured femur, with the rats divided into four experimental groups (*n* = 8 rats per group). These were the injury group: OVX + injury + non‐treatment, the PMMA group: OVX + injury + PMMA (20 μL), the PMMA gel group: OVX + injury + PMMA (4 μL) + gel (16 μL), and the PMMA‐spheroid gel group: OVX + injury + PMMA (4 μL) + gel (16 μL) + MSC spheroid (1 × 10^6^). Four weeks after the treatment injection, all rats were perfused as previously described.^41^, ^42^ Femurs/DRGs were extracted for an in vivo evaluation. In addition to the four experimental groups, three normal rats (without OVX/injury/treatment) and three OVX rats (without injury/treatment) were also subjected to an osteoporosis evaluation. All surgical interventions and postoperative animal care procedures were approved by the Institutional Animal Care and Use Committee (IACUC) of CHA University (IACUC210165).

### 4.10 Micro‐computed tomography

Shortly after sacrificing the rats, the femurs were safely separated using a bone cutter. The femurs were converted into digital imaging and communication in medicine files using μCT equipment (Quantum FX micro‐CT, PerkinElmer). We designated the VOI (21.56 mm^3^) per femur, and the femurs were reconstructed as 2D or 3D images. The BV (mm^3^) and tissue volume (TV, mm^3^) in the VOI in each case were measured using software (Analyze 12.0) included with the μCT equipment. BV (mm^3^), BV/TV (%), and the BMD (mg/cc) in the VOIs were quantified (*n* = 6 per group).

### 4.11 Tissue preparation for MTS and H&E staining

The femurs were post‐fixed with 10% neutral buffered formalin for 7 days and decalcified with 10% ethylene diamine tetra acetic acid (EDTA) for another 4 weeks. The EDTA solution was replaced once a week. The decalcified femurs were dehydrated, embedded in paraffin, serially sectioned (5 μm thick), and stained with MTS kits (IHC WORLD) or with H&E solution (BBC Biochemical).^43^ The H&E staining was performed as previously described.^44^ The H&E staining was performed as previously described.^38^ After MTS or H&E staining, the ROI (1800 × 1800 μm^2^) per femur was designated. The ROI area was designated as 100%, and the relative collagen‐stained area was calculated for the osteoporotic evaluation (normal rats versus ovariectomized rats). We also showed several bone parameters. OLCs, NBM, NB, and RBCs were demonstrated after H&E staining.

### 4.12 DRG sampling for immunofluorescence staining

Eight weeks after the OVX procedures, DRGs were extracted and stained with TRPV1/NeuN or iba1/NeuN antibodies via a method previously described.^45^ Detailed staining processes for the immunofluorescence (IF) images are explained in the Supporting Information. The ROIs (100 × 100 μm^2^) in a DRG were randomly designated (*n* = 8 per group). In the ROI, the TRPV1 intensity was divided into the NeuN intensity levels for quantification. For iba1 quantification, the ROI area was designated as 100% and the relative iba1 intensity was calculated. The IF intensity values were obtained at 20× magnification through a confocal laser‐scanning microscope (LSM 880). The values were quantified using the ImageJ program (National Institutes of Health, NIH).

### 4.13 Statistical analyses

All values are presented as the mean ± standard error of the mean (SEM). Tukey's tests were used to compare the significance among the groups. “*” *p* < 0.05, “**” *p* < 0.01, and “***” *p* < 0.001 were considered statistically significant.

## 5 CONCLUSION

We developed an injectable and biocompatible gel containing PMMA and MSC spheroids. The outcomes resulting from the treatment as part of this study show that the PMMA‐doped MSC spheroid gel can be useful for osteoporotic vertebral compression fracture patients.

## AUTHOR CONTRIBUTIONS

**Wan‐Kyu Ko:** Methodology (lead); validation (lead); visualization (equal); writing – original draft (lead). **Daye Lee:** Formal analysis (lead); investigation (lead); validation (equal); visualization (lead). **Seong Jun Kim:** Data curation (lead); investigation (equal). **Gong Ho Han:** Investigation (equal); methodology (equal). **Donghyun Lee:** Validation (equal). **Seung Hun Sheen:** Formal analysis (equal). **Seil Sohn:** Conceptualization (lead); funding acquisition (lead); supervision (lead); visualization (equal); writing – review and editing (lead).

## CONFLICT OF INTEREST STATEMENT

The authors declare no conflicts of interest.

## Supporting information


**DATA S1.** Supporting Information.

## Data Availability

The data that support the findings of this study are available from the corresponding author upon reasonable request.
