# Supplementary material for: Injection of a PMMA‐doped MSC spheroid gel for the treatment of painful osteoporotic vertebral compression fractures
Source: Bioeng Transl Med. 2023 Jul 16;8(6):e10577. doi: 10.1002/btm2.10577 (PMC10658584; doi:10.1002/btm2.10577)
Supplement: Supplementary file 1 — DATA S1. Supporting Information. [file BTM2-8-e10577-s001.docx]

Supporting Information

**Injection of a PMMA-doped MSC spheroid gel for the treatment of painful osteoporotic vertebral compression fractures**

Wan-Kyu Ko^1,2,a^, Daye Lee^1,2,a^, Seong Jun Kim^1,2^, Gong Ho Han^1,2^, Donghyun Lee ^3^, Seung Hun Sheen^1^, Seil Sohn^1*^

^1^Department of Neurosurgery, CHA Bundang Medical Center, CHA University, 59 Yatap-ro, Bundang-gu, Seongnam-si, Gyeonggi-do, 13496, Republic of Korea

^2^Department of Life Science, CHA University, Bundang-gu, Seongnam-si, Gyeonggi-do, Republic of Korea

^3^Preclinical Research Center, Daegu-Gyeongbuk Medical Innovation Foundation (DGMIF), Daegu, 41061, Republic of Korea

**Materials and methods**

**Preparation of polymerized PMMA and CHA gel**

The mixed volume ratio of PMMA-powder and MMA-liquid was 2:1 for the polymerization. The glycol chitosan (gC) was obtained from Sigma-Aldrich (MW: 50,000 Da). Sodium hyaluronate (HA, MW: 1,000,000 Da) was provided by Humedix (Anyang, Korea). The oxidized hyaluronate (oHA) was synthesized through an oxidation process of the HA using sodium periodate (NaIO_4_), as follows. HA (3.8 g) was dissolved in deionized water (360 ml). The sodium periodate (1.068 g) was dissolved in another amount of deionized water (40 ml). The dissolved sodium periodate was slowly added to the HA in a dark room. The mixture was stirred for 24 h and 1 mL of ethylene glycol (Sigma) was added to the mixture to neutralize the unreacted sodium periodate. The resultant solution was dialyzed for seven days using dialysis membranes (Spectrum Spectra, molecular weight cut off (MWCO): 12–14 K). After dialysis, the oHA solution was lyophilized. Two % weight per volume (w/v) of gC and 3% w/v of oHA were dissolved in separate amounts of Dulbecco's phosphate-buffered saline (DPBS, GIBCO, Life Technologies, Carlsbad, CA, USA). The volumetric ratio of the 2% gC and 3% oHA to form the gel was 9:1.

**Cell viability test**

The medium incubated with the four types of gels was changed with fresh medium containing the kit solution (500 μL volume of 0.1 mL/ml) for the cell viability test. The kit solution was sustained for 90 minutes in a cell incubator. Afterwards, the absorbance was evaluated with a microplate reader (Thermo) at 450 nm. The MSCs embedded in gels were stained with calcein-AM/EthD-1 of the Live/Dead staining kit. After reacting for 5 minutes, the MSCs in all groups were observed at 10 × magnification using a confocal laser-scanning microscope (LSM 880, Carl Zeiss, Jena, Germany).

**qRT-PCR**

The RNA was extracted using Trizol reagent (Invitrogen) according to the manufacturer's instructions. Complementary DNA (cDNA) was synthesized from 1 μg of total RNA using a Maxim RT Premix kit (iNtRON Biotechnology, Inc., Korea). The qRT-PCR step was performed with an ABI Step-One real-time PCR system (Applied Biosystems, Warrington, UK). The reaction mixture consisted of the SYBR Green 2X PCR Master Mix (Applied Biosystems), a cDNA template, and forward/reverse primers. The relative expression values of TGF-β, IL-10, Runx2, OSX, OPN, and Sost were normalized to that of GAPDH using the 2^-ΔΔCT^ method. The primers were obtained from Bioneer (Daejeon, Korea). The primer sequences used in this study are shown in Table S1.

**DRG sampling for IF staining**

The rats were anesthetized with an overdose of chloral hydrate and perfused with heparinized saline followed by 4% paraformaldehyde. DRG samples were sectioned at 5 μm thickness. According to standard procedures for IF staining, sections were treated with blocking solution to prevent nonspecific binding reaction for 1 h. Afterwards, they were stained by incubation overnight at 4 °C with the following primary antibodies: monoclonal mouse anti-mouse TRPV1 (1:500; Cat. BS397; R&D system)/anti-rabbit NeuN (1:500; Cat. ab177487; Abcam, Cambridge, UK) or anti-goat iba1 (1:200; Cat. ab5076; Abcam)/anti-mouse neuronal nuclear (NeuN, 1:100; Cat. MAB377; Merk Millipore, Milan, Italy). Afterwards, the sections were incubated with fluorescent secondary goat anti-mouse Alexa 488/donkey anti-mouse Alexa 647 (final dilution 1:2000, Invitrogen) or donkey anti-mouse Alexa 488/donkey anti-goat Alexa 647 (final dilution 1:1000, Invitrogen) for two hours at room temperature.

**Supporting Figures**

**Figure S1.** Cross-linking process between gC and oHA.

**Figure S2.** Mass measurement of the 8 (gel):2 (PMMA) ratio for 21 days.

**
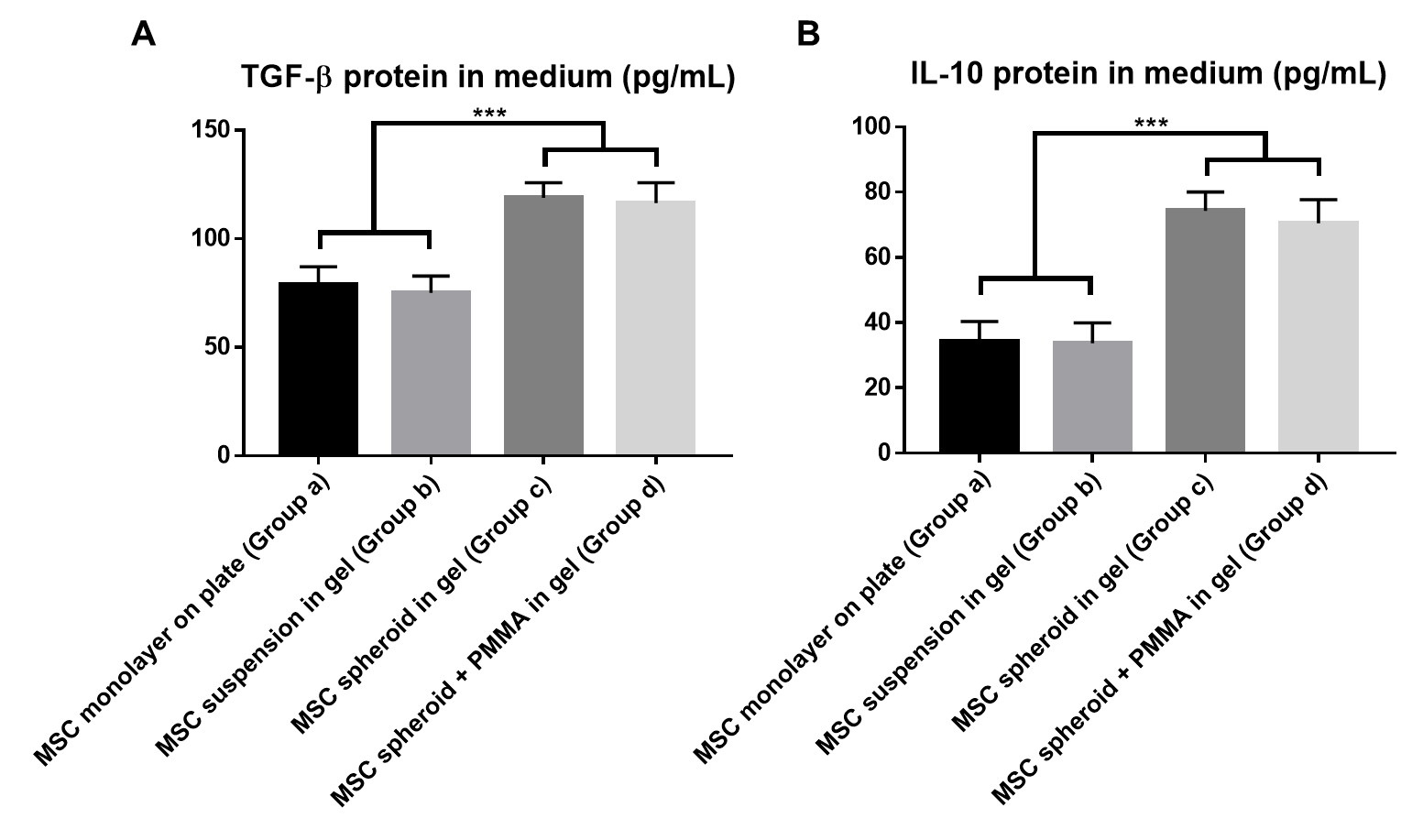
**

**Figure S3.** The (**A**) TGF-β and (**B**) IL-10 protein expression in MSCs cultured on plate or in gel. Multiple comparisons among the four groups were evaluated with a one-way analysis of variance (ANOVA). The results are expressed as the mean ± standard error of the mean (SEM, n = 4 per group): ***p < 0.001; significant differences among the four groups were shown.


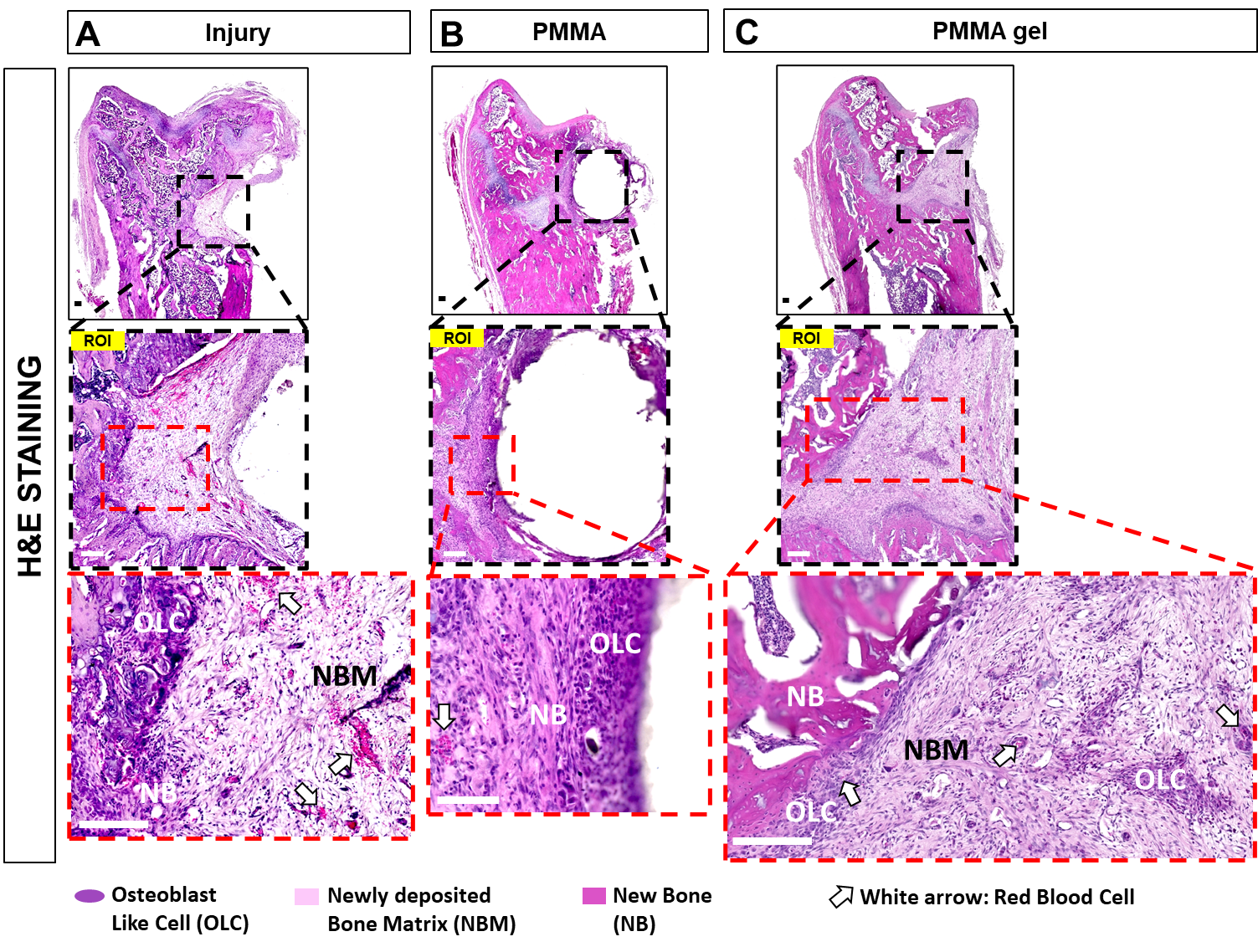


**Figure S4.** Histological evaluation. Representative H&E stained images for (**A**) Injury, (**B**) PMMA, and (**C**) PMMA gel (scale bar: 200 μm). A ROI (1,800 × 1,800 μm^2^) per femur was designated. Purple, deep pink, light pink, and red in the ROI indicate the osteoblast like cell (OLC), new bone (NB), newly deposited bone matrix (NBM), and red blood cell (RBC, designated with arrows), respectively.

**Supporting Table**

**Table S1. Nucleotide sequences of primers used in qRT-PCR.**

| **Gene** | **Forward (5' - 3')** | **Reverse (5' - 3')** |
| --- | --- | --- |
| **TGF-β** | **GCAACAACGCAATCTATGAC** | **CCTGTATTCCGTCTCCTT** |
| **IL-10** | **GCCTGCTCTTACTGGCTGGA** | **TCTGGCTGACTGGGAAGTGG** |
| **Runx2** | **GCCGGGAATGATGAGAACTA** | **TTGGGGAGGATTTGTGAAGA** |
| **OSX** | **CTGGGAAAAGGAGGCACAAAGA** | **GGGGAAAGGGTGGGTAGTCATT** |
| **OPN** | **TGAGACTGGCAGTGGTTTGC** | **CCACTTTCACCGGGAGACA** |
| **Sost** | **GGCAAGCCTTCAAGAATGATGCCA** | **TGTACTCGGACACGTCTTTGGTGT** |
| **GAPDH** | **GCAAGTTCAACGGCACAG** | **GCCAGTAGACTCCACGACA** |
